# Efficacy of venetoclax combined with hypomethylating agents in young, and unfit patients with newly diagnosed core binding factor acute myeloid leukemia

**DOI:** 10.1038/s41408-023-00928-1

**Published:** 2023-10-11

**Authors:** Keyuan Zhang, Xiang Zhang, Yang Xu, Shengli Xue, Huiying Qiu, Xiaowen Tang, Yue Han, Suning Chen, Aining Sun, Yanming Zhang, Depei Wu, Ying Wang

**Affiliations:** 1 National Clinical Research Center for Hematologic Diseases, Jiangsu Institute of Hematology, The First Affiliated Hospital of Soochow University, Suzhou, China; 2 Institute of Blood and Marrow Transplantation, Collaborative Innovation Center of Hematology, Soochow University, Suzhou, China; 3 Key Laboratory of Thrombosis and Hemostasis of Ministry of Health, Suzhou, China; 4 Department of Hematology, The Affiliated Huai’an Hospital of Xuzhou Medical University and The Second People’s Hospital of Huai’an, No 62, Huaihai Road (S.), Huai’an, China; 5 State Key Laboratory of Radiation Medicine and Protection, Soochow University, Suzhou, China

**Keywords:** Acute myeloid leukaemia, Combination drug therapy

**TO THE EDITOR:**

Core binding factor acute myeloid leukemia (CBF-AML) features the recurrent chromosomal rearrangements t(8;21)(q22;22) and inv(16)(p13.1q22)/t(16;16)(p13.1;q22), which encode the RUNX1::RUNX1T1 and CBFB::MYH11 fusion genes; CBF-AML is considered chemosensitive and classified within the favorable prognosis group [1]. Induction chemotherapy with cytarabine/anthracycline-based regimens and high-dose cytarabine (AraC)-based regimens is the standard of care for newly diagnosed (ND) or refractory/relapsed (R/R) CBF-AML, respectively [1, 2]. However, some patients are not eligible for these intensive therapies due to poor functional status and severe comorbidities.

Venetoclax (VEN) in combination with a hypomethylating agent (HMA) (azacitidine or decitabine) has emerged as a promising treatment for frail AML patients who are 75 years of age or older, or have medical conditions that prevent the use of standard intensive chemotherapy [3]. Previous studies have demonstrated an excellent remission rate with modest organ toxicities of the VEN + HMA regimen in both ND and R/R patients [3, 4]. However, there are limited data about the efficacy in patients with CBF-AML, because they have been excluded from most VEN-related studies. Therefore, in this study, we sought to investigate the feasibility of this combination as treatment of 30 newly diagnosed patients with poor functional status with CBF-AML. The study was approved by the institutional review board of our hospital, and informed consent was obtained.

Patients with CBF-AML who received VEN + HMA induction therapy between April 2019 and February 2023 at our center were retrospectively analyzed. Patients received azacitidine 75 mg/m^2^ for 7 days or decitabine 20 mg/m^2^ for 5 days, and venetoclax daily on days 1 through 28. The target dose of VEN was 400 mg daily but was reduced if coadministered with CYP3A inhibitors as recommended. The primary objective of this study was to evaluate the rate of objective response, including complete remission (CR), CR with incomplete count recovery (CRi), partial remission (PR) and morphologic leukemia-free state. Hematologic and molecular responses were assessed by analysis of bone marrow aspirates. Measurable residual disease (MRD) was monitored using real-time quantitative reverse transcriptase-polymerase chain reaction. The absolute copy numbers of fusion gene transcripts were normalized to those of ABL (expressed as copies per 10^5^ copies of ABL). Subsequent postinduction therapy varied at the discretion of each physician.

Thirty patients were included in this study (Supplementary Fig. 1). The reasons for ineligibility for intensive therapies were active pulmonary infection (21 patients), anal infection (2 patients), sepsis (2 patients), liver and renal disease (3 patients) and cardiac comorbidities (2 patients). The patient baseline characteristics are summarized in Table 1. Eighteen (60%) patients were men and 12 (40%) were women; the median age of the patients was 40 years (range, 15–68). Thirteen (43%) patients had t(8;21) cytogenetics and 17 (57%) patients had inv(16)/t(16;16) cytogenetics. The most common mutations were KIT mutations (16 patients (53%)), RAS mutations (10 patients (33%)), and FLT3 mutations (4 patients (13%)). No patient had mutations in TP53.

**Table 1:** Patient characteristics.

Characteristic *N* (%)/median [range]	All *N* = 30	t(8;21) *N* = 13	Inv(16)/t(16;16) *N* = 17	*P*
Age (years)	40 [15–68]	38 [25–68]	41 [15–60]	0.950
Sex				1.000
Male	18 (60)	8 (62)	10 (59)	
Female	12 (40)	5 (38)	7 (41)	
AML type				1.000
De novo	29 (97)	13 (100)	16 (94)	
Secondary	1 (3)	0 (0)	1 (6)	
WBC level(×10^9/L)	16.87 [1.22–200.02]	8.00 [2.10–49.52]	33.76 [1.22–200.02]	0.054
BM blasts(%)	50 [15–84]	43 [20–84]	56 [15–84]	0.589
Additional Cytogenetic abnormalities	17 (57)	9 (69)	8 (47)	0.283
Kinase mutations, No. (%)				
KIT	16 (53)	9 (69)	7 (41)	0.159
FLT3-ITD	1 (3)	1 (8)	0 (0)	0.433
FLT3 D835	3 (10)	2 (15)	1 (6)	0.565
RAS	10 (33)	2 (15)	8 (47)	0.119
TP53	0	0	0	
HMA type				0.465
Azacitidine	18 (60)	9 (69)	9 (53)	
Decitabine	12 (40)	4 (31)	8 (47)	

For 13 t(8;21) patients, CR/CRi was achieved in 4 patients (31%) after a single course of induction therapy. However, the MRD level at the end of cycle 1 was unsatisfactory, and the median transcript level of RUNX1::RUNX1T1 was 415,415 copies (range 600–1,004,990). Among 4 patients (31%) with PR, 2 patients were administered the second repeated course; of them, 1 achieved remission with RUNX1::RUNX1T1 33750 copies and 1 failed and died due to infection during reinduction with chemotherapy. The other 2 patients with PR were switched to traditional chemotherapy, and both achieved CR/CRi. The remaining 5 patients who failed the first course of VEN + HMA with no reduction in bone marrow blasts from baseline were salvaged with traditional chemotherapy, and all achieved CR/CRi. The 12 patients in remission were subsequently treated with consolidation chemotherapy with intermediate- or high-dose (ID/HD) AraC and 5 patients proceeded to allogeneic hematopoietic stem cell transplantation (allo-HSCT) due to persistent MRD.

For 17 inv(16)/t(16;16) patients, all patients achieved CR/CRi with a median transcript level of CBFB::MYH11 340 copies (range 0–20,650) after one cycle of treatment. All patients switched to ID/HD AraC-based consolidation chemotherapy. Three patients proceeded to allo-HSCT due to persistent MRD. One patient experienced hematologic relapse at 5 months after completion of chemotherapy and received reinduction with VEN + HMA. A second CR was achieved, and he underwent allo-HSCT thereafter.

Given the small patient number, it was hard to detect the value of mutational genotypes (Fig. 1a). However, in the t(8;21) group, no patient with KIT D816 mutations achieved CR/CRi after one cycle of therapy(4 patients in total, 2 PR, 2 NR), and the only 1 patient with FLT3-ITD failed the first course of VEN + HMA. As expected, the treatment was well tolerated, and all side effects were transient and reversible. No early death (within 30 days) was observed. The median duration of follow-up for the entire cohort was 11.6 months (range 2.0–39.0), and the 2-year probability of OS was 92.2% (95% CI, 70.8–98.0%) (Fig. 1b). Patients with inv(16)/t(16;16) had a trend toward a better 2‐year OS than patients with t(8;21) (100% vs. 81.5%, *p* = 0.090) (Fig. 1c).

**Fig. 1:**
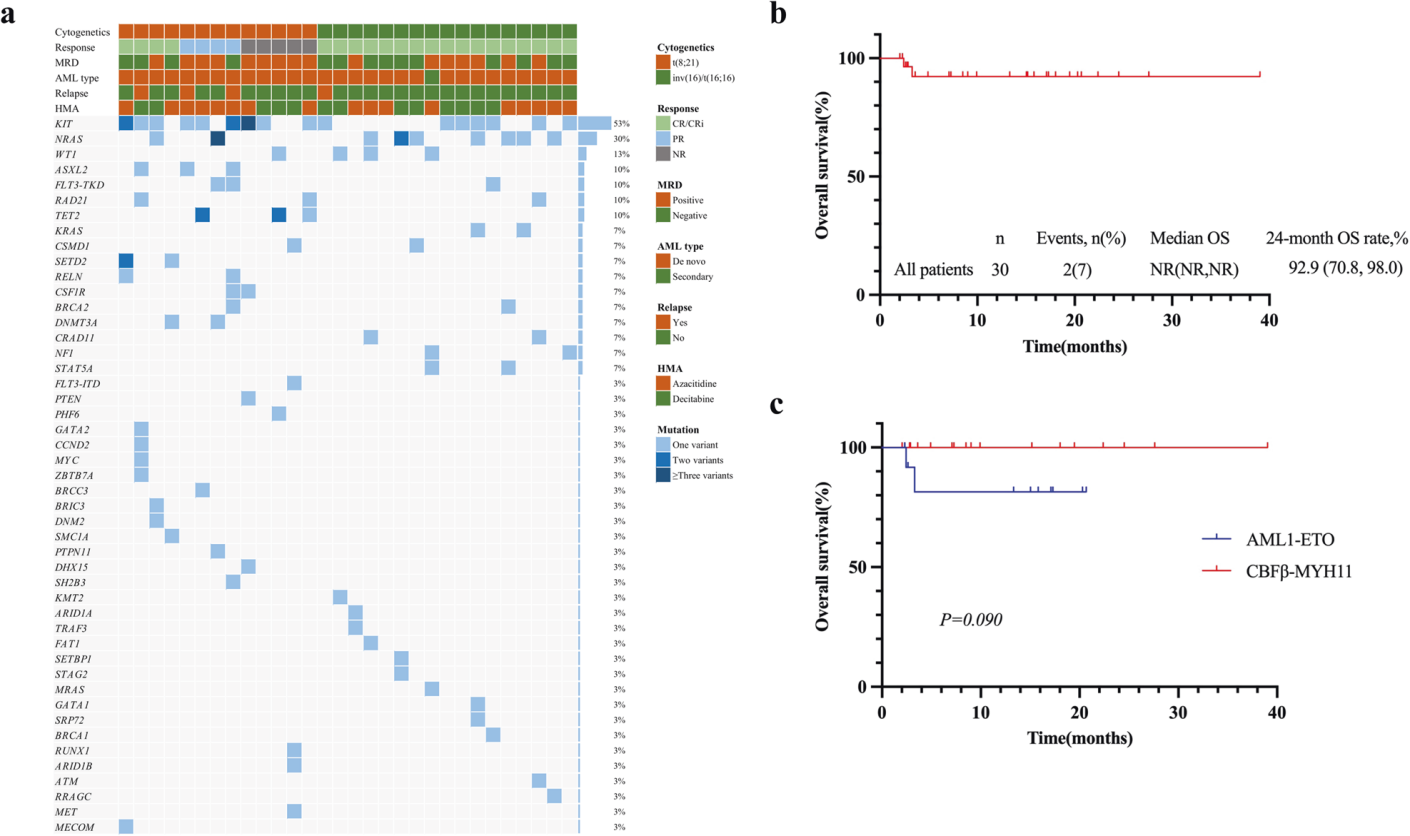
Clinical characteristics and response in 30 patients in this study. **a** Mutational landscapes. **b** Overall survival of all patients. **c** Overall survival stratified by cytogenetics.

Because of the exclusion criteria in VEN-based studies, data on VEN-HMA in CBF-AML patients are scarce. In a multicenter retrospective analysis of 46 patients with favorable-risk AML, Arslan et al reported that VEN-HMA resulted in CR/CRi rates of 88 and 70% in ND and R/R patients, respectively [5]. In that study, ten cases of CBF-AML were included, and the response rate was 80%, but the authors did not describe the patient details. To our knowledge, this is the largest study of the activity of VEN + HMA in CBF-AML. Our results show that the VEN + HMA regimen as induction therapy is a safe and effective treatment for inv(16)/t(16;16) AML; however, its efficacy in t(8;21) AML is suboptimal. Comparable to our data, in a recent analysis of VEN+azacitidine by Yu and colleagues that included 7 patients with t(8;21) (4 ND and 3 R/R), only 2 ND patients attained remission. Notably, the 5 patients who failed treatment all had KIT D816 mutation [6].

Though t(8;21) and inv(16)/t(16;16) AML are grouped together as CBF-AML and managed similarly, they are unique entities [1, 4]. Relapses are more frequent and long-term outcomes are usually worse in t(8;21) AML [1, 4]. Studies have also revealed that the genomic landscape is dramatically different between these two subtypes of CBF-AML, and mutations in chromatin modifiers and cohesins are more frequently observed in t(8;21) AML [7, 8]. More importantly, BCL-2 expression was found to be epigenetically silenced by aberrant transcription factor RUNX1::RUNX1T1 through inducing repressed chromatin configuration at its promoter [9]. Leukemia cells with t(8;21) may depend on antiapoptotic proteins other than BCL-2 [10]. All these phenomena may partially explain the inferior efficacy of VEN-HMA in t(8;21) AML.

On the other hand, in addition to the upregulation of alternative antiapoptotic proteins, other mechanisms of resistance to VEN-based therapy have been identified, such as inactivation of p53 protein and activating kinase mutations in proteins such as FLT3 and RAS [11]. Feasible strategies to mitigate or overcome resistance include novel combined BH3 mimetics inhibiting BCL-2/BCL-XL or BCL-2/MCL-1, increasing the dependence of leukemia cells on BCL-2 by inhibiting MCL1 and BCL-XL, and combinations of VEN with other mutation-targeted agents. Homoharringtonine was shown to have a synergistic antitumor effect with VEN-HMA by inducing stronger inhibition of MCL1/BCL-XL and increased activation of BCL-2 associated X, apoptosis regulator (BAX) in in vivo experiments. In a multicenter, phase 2 trial of VEN + azacitidine plus homoharringtonine in R/R AML, 6 patients with t(8;21) were included, and all achieved remission after treatment [12]. Similarly, we recently reported that midostaurin showed synergistic effects with VEN in an ex vivo study, and the combination therapy rescued two relapsed t(8;21) AML patients with KIT mutations who progressed rapidly after VEN + azacitidine therapy [13].

All 17 ND patients with inv(16)/t(16;16) who attained remission switched to consolidation with ID/HD Ara-C in our study. The reason is that VEN + HMA combination therapy needs to be administered indefinitely, and no plateau of the survival curve was observed in the VIALE-A trial [3]. However, considering that patients with favorable-risk AML, such as AML with NPM1 mutation, as well as patients with MRD negativity, appeared to have a good outcome with only VEN + HMA therapy (the median duration of response was not reached at a median follow-up of 15.1 months), the transcript level of CBFB::MYH11 was dramatically decreased, and MRD negativity was achieved in some patients after only one cycle of treatment in our study, the use of VEN + HMA as an alternative therapy for ID/HD AraC, especially in patients with older age or complications and MRD-negative CR, warrants future investigation. Furthermore, as there is growing interest in reevaluating the role of maintenance therapy with novel agents in AML [14], the application of VEN + HMA in inv(16)/t(16;16) AML deserves further study. In contrast, caution should be applied in t(8;21) AML.

In contrast to a former study by Maiti and colleagues, which showed that patients who had European Leukemia Net (ELN) intermediate or adverse risk AML and had R/R disease after front-line VEN-based therapy displayed high-risk disease biology and particularly poor survival (the median OS after VEN + HMA failure was only 2.4 months) [15], in this study, patients with t(8;21) AML with refractory disease after front-line VEN + HMA displayed a normal survival rate compared to historical data. Therefore, insensitivity to VEN-based therapy may not predict significantly inferior survival in t(8;21) AML.

In summary, despite the retrospective nature of and relatively small number of patients in this study, our data suggest that t(8;21) is a predictive biomarker of response in AML patients who are likely to achieve minimal benefit from VEN + HMA therapy, and AML patients with t(8;21) should be treated with other regimens. Conversely, VEN + HMA is a useful regimen for inv(16)/t(16;16) AML. Meanwhile, for elderly patients who are ineligible for intensive chemotherapy or allo-HSCT, future studies to evaluate the long-term efficacy of VEN + HMA is warranted.

### Supplementary information

Supplementary Figure 1 legend

Supplementary Figure 1

## Data Availability

The data that support the findings of this study are available from the corresponding author upon reasonable request.
