# Supplementary material for: Efficacy of venetoclax combined with hypomethylating agents in young, and unfit patients with newly diagnosed core binding factor acute myeloid leukemia
Source: Blood Cancer J. 2023 Oct 11;13(1):155. doi: 10.1038/s41408-023-00928-1 (PMC10567686; doi:10.1038/s41408-023-00928-1)
Supplement: Supplementary file 1 — Supplementary Figure 1 legend [file 41408_2023_928_MOESM1_ESM.docx]

Supplementary Figure 1. Flow diagram of the patients.
