# Supplementary figures and images for: Efficacy of venetoclax combined with hypomethylating agents in young, and unfit patients with newly diagnosed core binding factor acute myeloid leukemia
Source: Blood Cancer J. 2023 Oct 11;13(1):155. doi: 10.1038/s41408-023-00928-1 (PMC10567686; doi:10.1038/s41408-023-00928-1)

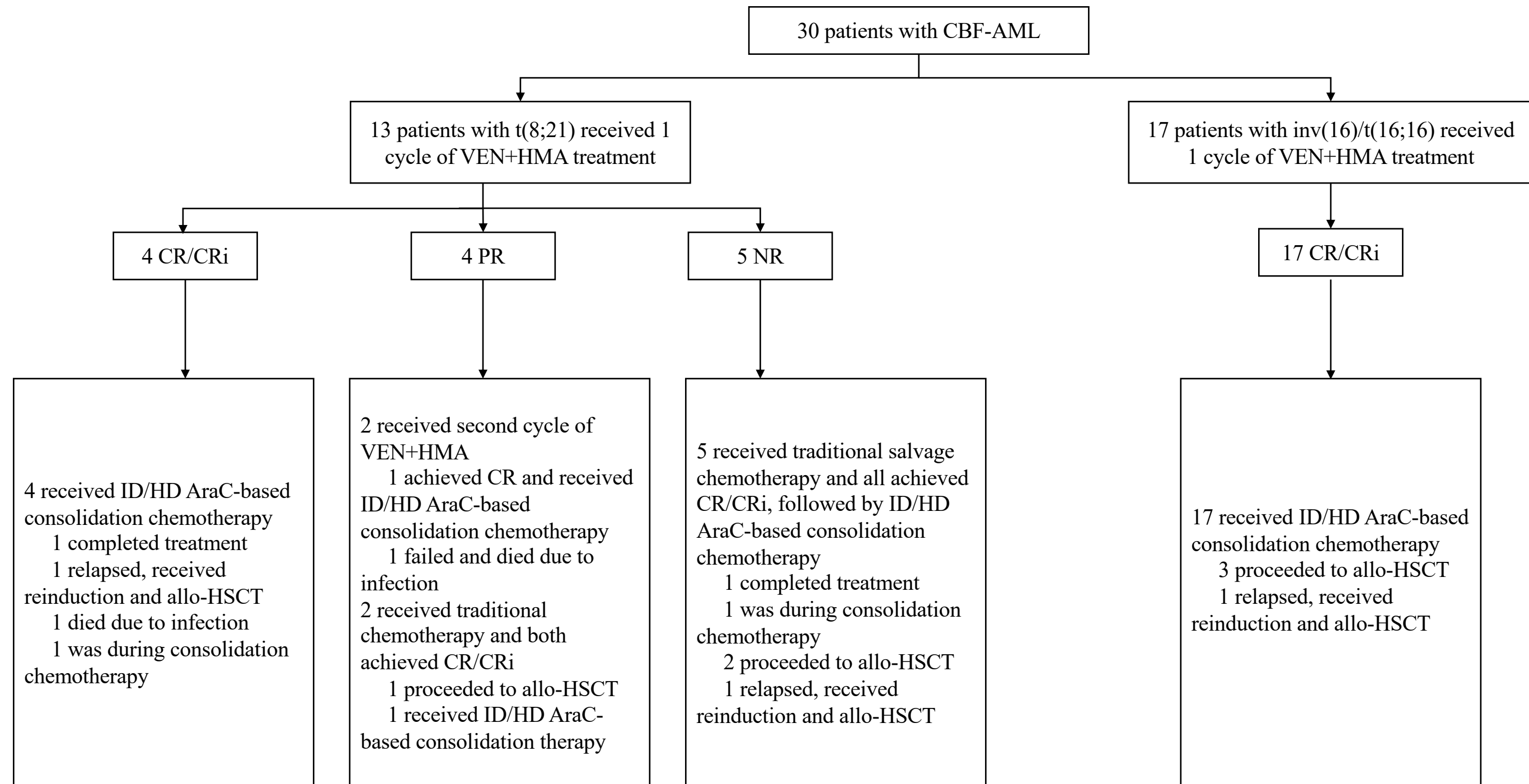

Supplement: Supplementary file 2 — Supplementary Figure 1 [file 41408_2023_928_MOESM2_ESM.pdf]
